# Herba Artemisiae Capillaris Extract Prevents the Development of Streptozotocin-Induced Diabetic Nephropathy of Rat

**DOI:** 10.1155/2018/5180165

**Published:** 2018-02-14

**Authors:** Jianan Geng, Xiaoyan Yu, Chunyu Liu, Chengbo Sun, Menghuan Guo, Zhen Li, Yingli Jin, Yinggang Zou, Jinghua Yu

**Affiliations:** ^1^Institute of Virology and AIDS, The First Hospital of Jilin University, Jilin University, Dongminzhu Street 519, Changchun 130000, China; ^2^Department of Experimental Pharmacology and Toxicology, School of Pharmacy, Jilin University, Fujin Road 1266, Changchun 130021, China; ^3^Acupuncture Department, The Affiliated Hospital to Changchun University of Chinese Medicine, 1478 Gongnong Road, Changchun 130021, China; ^4^Department of Pharmacology, College of Basic Medical Science, Jilin University, Xinmin Street 126, Changchun 130021, China; ^5^Department of Obstetrics Gynecology, The Second Hospital of Jilin University, Ziqiang Street 218, Changchun 130041, China

## Abstract

Diabetic nephropathy (DN) is a major cause of end-stage renal disease throughout the world; until now there is no specific drug available. In this work, we use herba artemisiae capillaris extract (HACE) to alleviate renal fibrosis characterized by the excessive accumulation of extracellular matrix (ECM) in rats, aiming to investigate the protective effect of the HACE on DN. We found that the intragastric treatment of high-dose HACE could reverse the effect of streptozotocin not only to decrease the level of blood glucose and blood lipid in different degree but also further to improve renal functions. It is worth mentioning that the effect of HACE treatment was comparable to the positive drug benazepril. Moreover, we found that HACE treatment could on one hand inhibit oxidative stress in DN rats through regulating enzymatic activity for scavenging reactive oxygen species and on the other hand increase the ECM degradation through regulating the activity of metalloproteinase-2 (MMP-2) and the expression of tissue transglutaminase (tTG), which explained why HACE treatment inhibited ECM accumulation. On the basis of above experimental results, we conclude that HACE prevents DN development in a streptozotocin-induced DN rat model, and HACE is a promising candidate to cure DN in clinic.

## 1. Introduction

Diabetes mellitus is a group of metabolic disorders in which there are high blood sugar levels over a prolonged period, and the incidence of diabetes in the world is increasing because of high sugar intake, high-fat diet intake, lack of physical exercise, and so on [1]. Chronic high blood sugar leads to the structural and functional lesion of multiple tissues, including the retinopathy, peripheral neuropathy, diabetic cardiomyopathy and kidney injury, and even tissue failure [2–5]. Among those complications, one of the most serious is diabetic nephropathy (DN), which is mainly characterized by accumulation of extracellular matrix (ECM) in glomeruli, as well as other secondary features: glomerular hypertrophy, proteinuria, renal fibrosis, and even renal damage [6, 7]. Now DN has become a global health problem; although the level of clinical nursing for DN patients has been greatly improved in recent years, the number of DN patients at end stage is still increasing year by year [8]. Therefore, studies should be conducted for better understanding of pathogenic mechanisms and novel therapeutic agents of DN.

Oxidative stress is involved in the pathogenic mechanisms of DN. Reactive oxygen species (ROS) are generated by sustained high glucose challenge, which are found by different teams [9, 10]; then ROS induce releasing of series fibrosis factor, promoting ECM remodeling and finally leading to renal interstitial fibrosis and damage [11, 12]. Meanwhile at DN stage, inhibiting the degradation of ECM usually leads to the accumulation of ECM in glomeruli, while matrix metalloproteinases (MMPs) system is responsible for the degradation of ECM [13, 14]. Under DN, state plasminogen activator inhibitor (PAI-1), which is the main inhibitor of activating plasminogen, is upregulated to inhibit the plasminogen activities and then to decrease the activities of plasmin and further subsequently to decrease the activates of plasmin-dependent MMPs [15]. Numerous studies have indicated that PAI-1 has a profound effect on the development of DN, and the symptom of DN would be significantly retarded after PAI-1 gene is knocked out [16]. Moreover, tissue inhibitor of metalloproteinases (TIMPs) can inhibit the activities of MMPs by noncovalent bonding to the activated MMPs on the proportion of 1 : 1, and TIMPs can inhibit the activation of MMP by combining with plasminogen [17]. Among the MMPs family, MMP-2 as well as its inhibitor TIMP-2 has been identified as the crucial mediator of ECM accumulation in the DN kidney [18]. Apart from MMPs and their regulators, tissue transglutaminase (tTG) can couple with fibronectin, collagen, and collagen peptide through covalent cross-linking effect, which are responsible for the widespread of ECM proteins to promote ECM accumulation [19, 20].

Herba artemisiae capillaris (HAC) is a traditional Chinese herbal medicine that is usually used in the liver protection, and it has remarkable therapeutic and protective effect for liver fibrosis [21], oxidative damage to liver [22], and any other liver injury [23], which were characterized by the excessive accumulation of ECM [24]. Meanwhile early research showed that HAC had a similar hypoglycemic effect to biguanide drugs in alloxan-induced mice [25]. But so far the effect of HAC extract (HACE) on DN has not been clear. This study is to provide theoretical basis for the treatment of DN with HACE and detect its possible mechanism from two aspects, the level of oxidative stress and regulating the ECM synthesis and degradation. This study provides a novel agent for a more specific therapy for DN.

## 2. Materials and Methods

### 2.1. Drug

The herba artemisiae capillaris extract (HACE) was supplied by Wuhan Yuancheng Gongchuang Technology Co., Ltd. (Wuhan, China). The extract ratio is 10 : 1 through water extract method; the contents of chlorogenic acid and p-hydroxyacetophenone in HACE were detected with HPLC. According to the Chinese Pharmacopoeia (2015), the chlorogenic acid content in HACE is 1.32% (>1.0%) and the p-hydroxyacetophenone content in HACE is 0.18% (>0.10%); its loss on drying is 4.6% (≤5.0%) and on ash it is 3.1% (≤5.0%). Heavy metals' purity is ≤10 ppm, arsenic ≤ 2 ppm, pentachloronitrobenzene (PCNB) ≤ 0.1 ppm, and aflatoxin ≤ 5 ppb, and the number of microbes is qualified. The drug was sealed and kept in a shady and dry place.

### 2.2. Animals

Wistar rats (male, weighing 200–220 g) were obtained from the Experimental Animal Center of Jilin University. The rats were housed in a pathogen-free facility with free access to the standard dried chow diet and water throughout the study. All the animal experiments were conducted following internationally recognized guidelines on animal welfare; moreover, this study was conducted in accordance with the Declaration of Helsinki, and the protocol was approved by the Ethics Committee at the First Hospital of Jilin University, and all animal experiments were approved by the Animal Care and Use Committee at the First Hospital of Jilin University.

### 2.3. Induction of Diabetes and Experimental Design

After adaptive feeding for a week, Wistar rats were fasted for 12 h and then received intraperitoneal single injection with 55 mg/kg streptozotocin (Sigma, St. Louis, MO, USA) in citrate solution (0.1 M citric acid, pH 4.5, and 0.2 M sodium phosphate, pH 4.5). Normal rats as control (Con group, *n* = 10) were given citrate buffer alone. One week later, the rats with blood glucose levels between 13.8 and 25 mmol/L and urine glucose levels ≥ 3+ as diabetic group were used following experiments. Then, the diabetic rats were randomly intragastrically given low-dose HACE (2 g/kg, HACE-L group, *n* = 10) and high-dose HACE (5 g/kg, HACE-H group, *n* = 10) for 12 weeks; diabetic rats treated with benazepril (Beijing Novartis Pharma Co., Ltd., Beijing, China) were used as a positive control (75 mg/kg, Bena group, *n* = 10). Control group was treated with vehicle control (0.5% CMC-Na) for 12 weeks.

### 2.4. Measurement of Blood Glucose, Blood Lipids, and Renal Function

The rats were sacrificed at 12 weeks after HACE treatment. Getting the blood plasma for detecting blood glucose, getting serum for detecting blood urine nitrogen, creatinine, and blood lipids including total cholesterol, triglyceride, low-density lipoprotein-cholesterol, and high-density lipoprotein-cholesterol, and getting the urine in the last 24 h for the detection of albuminuria, all samples were measured according to the instruction utilizing a Hitachi 7150 Biochemical Autoanalyzer (Hitachi, Tokyo, Japan).

### 2.5. Antioxidation Assays

In this experiment, the activities of Cu/Zn superoxide dismutase (SOD), hydrogen peroxidase (CAT), and glutathione peroxidase (GSH-PX) and content of malondialdehyde (MDA) were analyzed by reagent kit purchased from Nanjing Jiancheng Bioengineering Institute (Nanjing, China) according to the instruction.

### 2.6. Histological Examinations

One portion of kidney tissues was fixed in 10% buffered formalin and embedded in paraffin for a light microscopic study (Nikon, Japan). The degrees of mesangial matrix expansion in different groups of rats were determined as periodic acid-Schiff (PAS) reagent positive staining in the mesangial region; the glycogen and mucus proteins deposited on mesangial area were dyed deep purple for PAS positive staining and the cell nuclei were dyed blue. In addition, we observed the collagen fibrils content in kidney through Masson staining; the collagen fibrils in kidney tissue were stained by aniline blue. Staining results were all observed under the microscope and photographed and analyzed utilizing Image-Pro Plus 6 analysis software in a blinded manner.

### 2.7. Immunohistochemistry

The protein levels of fibronectin, type IV collagen, PAI-1, TIMP-2, and tTG in renal tissue sections were examined by immunochemistry. Briefly, the renal tissue sections were treated with mouse anti-fibronectin monoclonal antibody, mouse anti-type IV collagen monoclonal antibody (Santa Cruz Biotechnology, Santa Cruz, CA), mouse anti-tTG monoclonal antibody (Lab Vision, USA), and rabbit anti-PAI-1 polyclonal antibody and rabbit anti-TIMP-2 polyclonal antibody (Proteintech Group, China); then samples were incubated with horseradish peroxidase- (HRP-) anti-rabbit IgG or HRP-anti-mouse IgG (Santa Cruz Biotechnology, Santa Cruz, CA) and then reacted with diaminobenzidine (DAB); after that samples were counterstained with hematoxylin. The percentages of positive staining areas in the glomerulus were determined semiquantitatively using Image-Pro Plus 6 analysis system.

### 2.8. Western Blot Assays

Renal cortical tissues of rats were sliced in different groups and, respectively, homogenized in lysis buffer and the supernatants were collected. Following quantification of protein concentrations, the tissue lysates were separated by SDS-PAGE on a 10% polyacrylamide gel, followed by transferring onto nitrocellulose membranes (Millipore, Massachusetts, USA). The membranes were incubated with rabbit anti-PAI-1 polyclonal antibody (1 : 1000 dilution, Proteintech, Wuhan, China), anti-TIMP-2 polyclonal antibody (1 : 1000 dilution, Proteintech, Wuhan, China), and mouse anti-tTG monoclonal antibody (1 : 300 dilution, Lab Vision, USA) at 4°C overnight. The bound antibodies were severally detected with HRP-conjugated anti-rabbit IgG or anti-mouse IgG and after that visualized using an enhanced chemiluminescence kit, according to the manufacturer's instructions (Thermo, USA). The relative levels of target proteins to control GAPDH were determined by densimetric scanning.

### 2.9. Gelatin-Zymography

Renal cortical tissues of rats were sliced for 30 mg in different groups and, respectively, homogenized in cool buffer (100 mM Tris-Cl, 1 mM CaCl2, pH 7.4) for fully sonic disruption about 10 seconds and then centrifuged and the supernatants were collected for detection. Then the samples were separated by 10% SDS-PAGE with 1 mg/mL gelatin followed by mixing the supernatants with 1% Triton X-100 (1 : 10). After electrophoresis, the gelatum was rinsed in rinse buffer (2.5% Triton-100, 50 mM Tris, 200 mM NaCl, 5 mM CaCl2, 1 *μ*M ZnCl2, and 0.02% NaN3, pH 7.6) for 3 times and incubated overnight. Next, the gelatum was stained with Coomassie Brilliant Blue and decolorized with corresponding buffer which contains different concentrations of methanol and acetic acid. At last, the enzyme amount was analyzed with statistical method.

### 2.10. Statistical Analyses

Data are presented as mean ± SD. The difference among the five groups was analyzed by one-way ANOVA and post hoc analysis by Bonferroni correction. The difference between two groups was analyzed by Student's *t*-test using SPSS 19.0 software. ^*∗*^*P* values less than 0.05 were considered to represent a statistically significant difference.

## 3. Results

### 3.1. HACE Reduced Blood Glucose Levels and Improved Renal Functions of Rats with Diabetic Nephropathy

**Figure 1:**
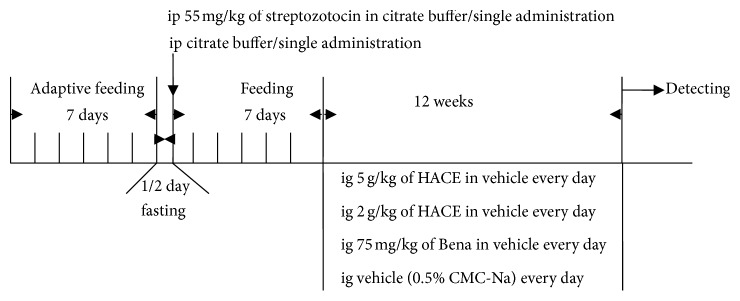
The time schedule of this study.

In the present study, intraperitoneal single injection of streptozotocin was done for inducing diabetic nephropathy (DN), and HACE or benazepril was administrated for the DN treatment; the time schedule was shown in Figure 1. It was found that the glucose value after intraperitoneal injection of streptozotocin was 24.07 ± 1.35 mmol/L, which was nearly three times of that in the control group (8.20 ± 1.35 mmol/L), and it was higher than the maximum normal value of blood glucose (13.8 mmol/L), indicating that diabetic model in rat was successful. Low-dose HACE treatment did not reduce the blood glucose level of diabetic rat, while high-dose HACE treatment showed significant reduction of the blood glucose level from 24.07 ± 1.35 mmol/L to 15.08 ± 11.80 mmol/L (*P* < 0.05, Table 1). Therefore, high-dose HACE treatment could lower the blood glucose level in diabetic model, and the results were the same as those of Pan et al.'s study [25].

To examine the renal injury and dysfunction induced by a single administration of streptozotocin and whether HACE could improve renal function of diabetic rats or not, indicators of renal function were detected in urine samples, including blood urea nitrogen, creatinine, and albuminuria. Among the indicators, blood urea nitrogen and creatinine were not affected by streptozotocin injection compared to control group; however, albuminuria, which was the protein albumin abnormally present in the urine, was significantly elevated in the diabetic model group compared with the control group (Table 1), which further indicated that the diabetic rats had suffered from renal dysfunction, and the streptozotocin-induced DN model had been successfully established. More importantly, after high-dose HACE treatment, the albuminuria was declined from 0.50 ± 0.15 mg to 0.34 ± 0.15 mg (*P* < 0.05), which was parallel to positive drug treatment (0.34 ± 0.13 mg, *P* < 0.05, Table 1). Therefore, HACE could improve the renal function of rat with DN.

DN is characterized by not only hyperglycemia but also endocrine disorder, because persistent hyperglycemia can induce serious metabolic disturbance including lipid metabolism [26, 27]; therefore, the levels of total cholesterol, triglyceride, low-density lipoprotein-cholesterol, and high-density lipoprotein-cholesterol which were related to blood lipid level and lipid metabolism were detected. It was found that low-density lipoprotein-cholesterol of diabetic rats (*P* < 0.01) and total cholesterol (*P* < 0.05) were obviously increased compared to control group, indicating that in DN rats abnormal lipid level and lipid metabolism occurred. However, followed by treatment with high-dose HACE or positive control drug benazepril, total cholesterol was decreased compared to DN group (*P* < 0.01, Table 1). Therefore, HACE could regulate the lipid level and lipid metabolism.

There was no significant difference in the content of blood urea nitrogen, creatinine, triglyceride, and high-density lipoprotein-cholesterol in DN rats compared to control rats; the symptoms of DN rat model induced by streptozotocin are not totally consistent with DN patients in clinic. More importantly, high-dose HACE treatment decreased the level of triglyceride compared to DN group and even control group (Table 1), indicating that HACE not only had renal function protective effect but also could decrease the level of lipid.

In summary, the protective effect of high dosage of HACE on the kidney was comparable to the positive drug benazepril, and HACE had no obvious side effect.

### 3.2. HACE Inhibited ECM Accumulation in Glomeruli of Rats with DN

**Figure 2:**
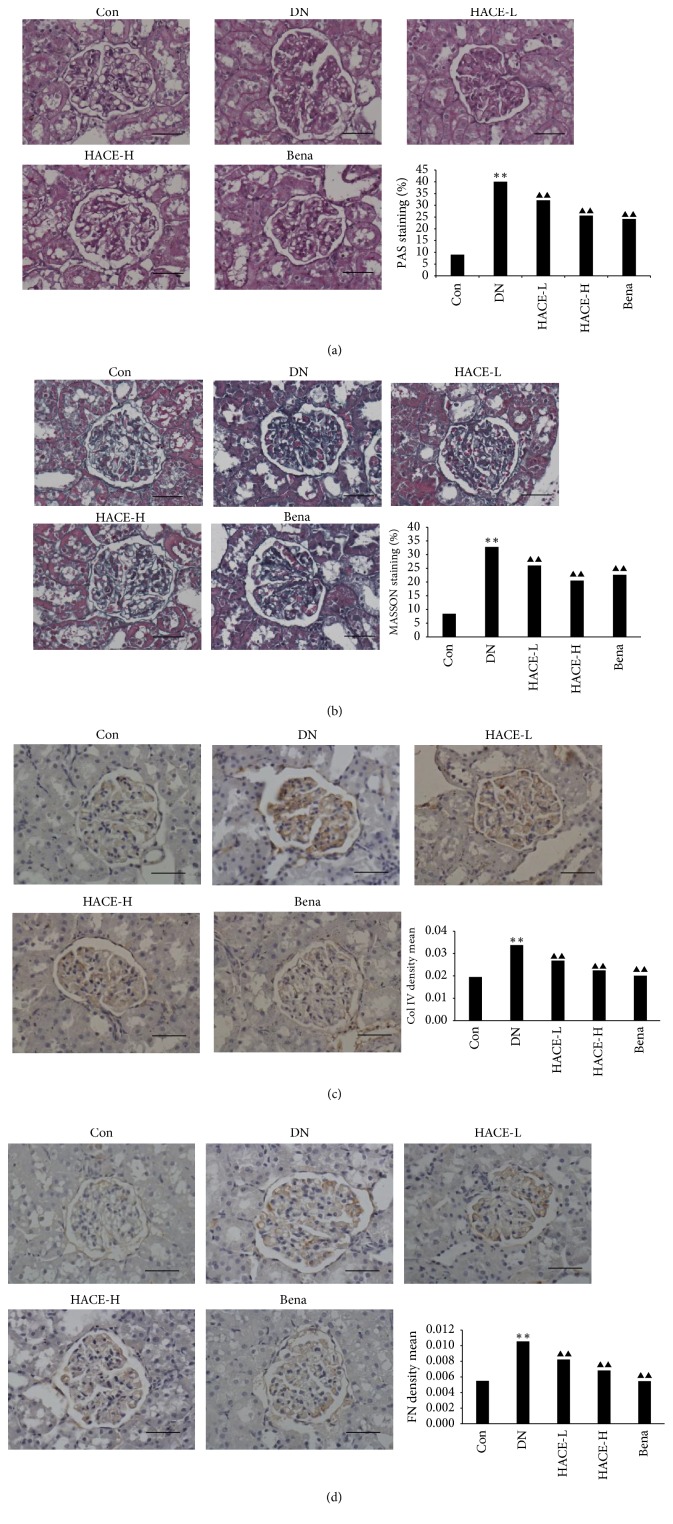
The effect of HACE treatment on the ECM accumulation in the diabetic kidney. ECM accumulation in the kidney was measured by PAS staining (a) and Masson staining (b) under a light microscope (×400). Quantitative analysis for the percentage of PAS and Masson in the glomerulus by the computer image analysis system is presented as the histogram. ((c) and (d)) Protein levels of type IV collagen (c) and fibronectin (d) as the two main components of ECM were detected by immunohistochemistry. Positive staining is shown in brown. Sections are counterstained with hematoxylin (magnification: ×400). Bar = 100 *μ*m. Quantitative results of these stain photos are shown in the histogram, which represents the density mean (density mean = IOD/area). Each bar represents mean ± SD (*n* = 10). Con: nondiabetic control group; DN: streptozotocin-induced diabetic group; HACE-L: low-dose-HACE-treated group; HACE-H: high-dose-HACE-treated group; Bena: benazepril; Col IV: type IV collagen; FN: fibronectin. ^*∗∗*^*P* < 0.01 versus Con; ^▲▲^*P* < 0.01 versus DN.

ECM accumulation is one character of pathological alterations in glomeruli of DN patients and animal models [2]. In the present study, the phenomenon of ECM accumulation was evaluated by the periodic acid-Schiff (PAS) reagent and Masson staining, respectively. In PAS staining, increased glomerulus, mesangial hyperplasia, thick basement membrane, and accumulation of ECM substances such as glycogen and mucus proteins (dyed deeply prunosus) were observed in the mesangial region of the streptozotocin-induced DN group compared to control group (Figure 2(a)). And, in the Masson staining, the deposition of collagen fiber (dyed blue) was found in DN rats compared to control group (Figure 2(b)), indicating that streptozotocin induced the obvious renal damage. At the same time, it was observed that high-dose HACE treatment reversed the renal damage induced by streptozotocin, and the effect of high-dose HACE treatment was similar to the positive drug benazepril (Figures 2(a) and 2(b), *P* < 0.01).

It was reported that type IV collagen and fibronectin were the predominant components of ECM and they were generally considered as the predictors of ECM accumulation [7], and this study found that the expressions of type IV collagen and fibronectin were distinctly upregulated in the DN group (*P* < 0.01), while expressions of type IV collagen (Figure 2(c)) and fibronectin (Figure 2(d)) in glomeruli were significantly reduced by HACE treatment (*P* < 0.01); in particular high-dose HACE had comparable effect to positive drug benazepril. These results indicated that HACE could inhibit the ECM accumulation in glomeruli of DN rats.

### 3.3. HACE Relieved Oxidative Stress in Glomeruli of Rats with DN

**Figure 3:**
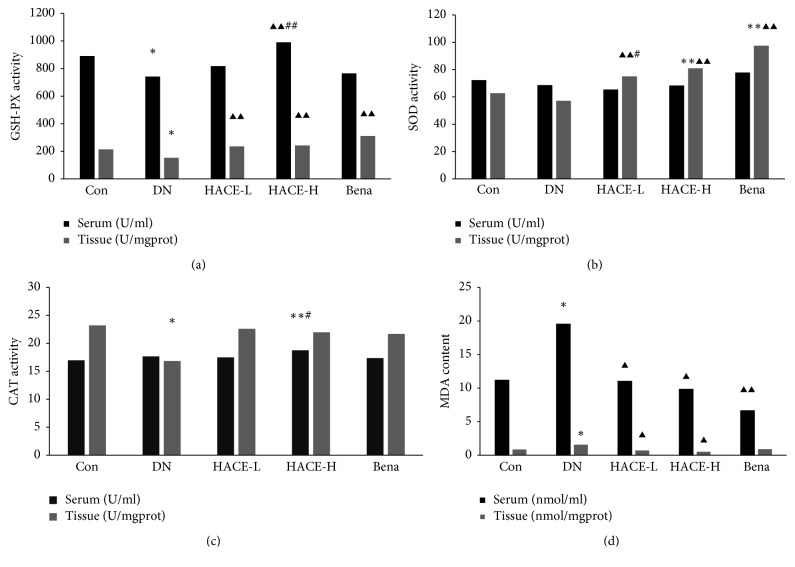
The effect of HACE treatment on the activity of GSH-PX, SOD, CAT, and MDA. (a) GSH-PX activity. (b) SOD activity. (c) CAT activity. (d) MDA activity. Con: nondiabetic control group; DN: streptozotocin-induced diabetic group; HACE-L: low-dose-HACE-treated group; HACE-H: high-dose-HACE-treated group; Bena: benazepril; SOD: superoxide dismutase; CAT: hydrogen peroxidase; GSH-PX: glutathione peroxidase. Data are presented as mean ± SD from 10 animals (*n* = 10) for each group. ^*∗*^*P* < 0.05 versus Con; ^*∗∗*^*P* < 0.01 versus Con; ^▲^*P* < 0.05 versus DN; ^▲▲^*P* < 0.01 versus DN; ^#^*P* < 0.05 versus Bena; ^##^*P* < 0.05 versus Bena.

To elucidate whether oxidative stress was involved in the progression of renal dysfunction and morphological alterations in streptozotocin-induced DN rats and confirm the potential inhibitory effect of HACE on oxidative stress, the activity of superoxide dismutase (SOD), hydrogen peroxidase (CAT), and glutathione peroxidase (GSH-PX), whose main biological role was to protect the organism from oxidative damage, was detected in serum and glomeruli for analyzing systemic and local antioxidant activity, respectively. Firstly, the activity of GSH-PX was remarkably decreased in serum and glomeruli in DN group compared to control group (*P* < 0.05), indicating that DN was accompanied with the loss of GSH-PX activity, while low-dose HACE (*P* < 0.01), high-dose HACE (*P* < 0.01), or benazepril (*P* < 0.01) treatment significantly upregulated GSH-PX activity in glomeruli, and only high-dose HACE increased the activity of GSH-PX in serum compared to DN group (*P* < 0.01, Figure 3(a)). Therefore, HACE treatment significantly improved the systemic and local activity of GSH-PX, and high-dose HACE treatment had better effect than positive drug treatment. Secondly, SOD activity was analyzed; it was found that the activity of SOD in DN group was similar to control treatment group in either serum or glomeruli, indicating that the SOD activity was not decreased in DN model, but low-dose HACE, high-dose HACE, or positive drug benazepril treatment could improve the activity of SOD in glomeruli (*P* < 0.01, Figure 3(b)); therefore HACE and benazepril could regulate the SOD activity to clean glomerulus reactive oxygen species. Thirdly, CAT activity was analyzed, and it was found that the activity of CAT was decreased in glomeruli in DN model compared to control group (*P* < 0.05), but low-dose HACE, high-dose HACE, or positive drug benazepril only has trend to increase the activity of CAT; meanwhile importantly it was found that in serum high-dose HACE treatment increased the CAT activity which was even higher than control group (Figure 3(c)); therefore HACE treatment increased the systemic CAT activity to scavenge reactive oxygen species. Finally, the level of MDA was detected; it was found that MDA level was increased in either glomeruli or serum of diabetic model compared to control treatment (*P* < 0.05), and high-dose or low-dose HACE treatment decreased the level of MDA compared to DN model group in either glomeruli or serum (*P* < 0.05), but positive drug benazepril only decreased the level of serum MDA (*P* < 0.01), not glomeruli (Figure 3(d)). The results suggested that HACE had an antioxidant activity and could relieve the oxidative stress in serum and glomerular of streptozotocin-induced DN rats.

### 3.4. HACE Enhanced the MMP-2 Activity in Glomeruli of Rats with DN

**Figure 4:**
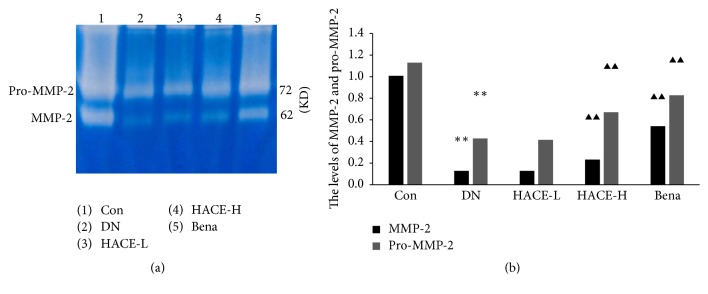
The activity of MMP-2. (a) The activity of MMP-2 was detected by zymography; MMP-2 will hydrolyze the gelatin at corresponding position and reveal transparent visible band under blue background; the brightness of band represents the activity of the enzyme. 62 KD brand is the active form of MMP-2; 72 KD brand is the form of pro-MMP-2. (b) The relative value of MMP-2 and pro-MMP-2 activity. Enzyme volume = strip area × (band gray − background gray). The results were standardized to MMP-2 enzyme volume and normalized to 1.0 in control group. Con: nondiabetic control group; DN: streptozotocin-induced diabetic group; HACE-L: low-dose-HACE-treated group; HACE-H: high-dose-HACE-treated group; Bena: benazepril. ^*∗∗*^*P* < 0.01 versus Con; ^▲▲^*P* < 0.01 versus DN.

MMP-2 had been recognized as a major enzyme promoting degradation of ECM [9]; it was found that, compared with control group, the MMP-2 (62 KD) activity and the level of pro-MMP-2 (72 KD) were distinctly decreased (*P* < 0.01, Figures 4(a) and 4(b)) in glomeruli of DN rats. After high-dose HACE or benazepril treatment, compared with DN group, the MMP-2 activities and the level of pro-MMP-2 were distinctly increased (*P* < 0.01, Figures 4(a) and 4(b)). Therefore, HACE could regulate the MMP-2 activity.

### 3.5. HACE Decreased the Expression of TIMP-2 in Glomeruli of Rats with DN

**Figure 5:**
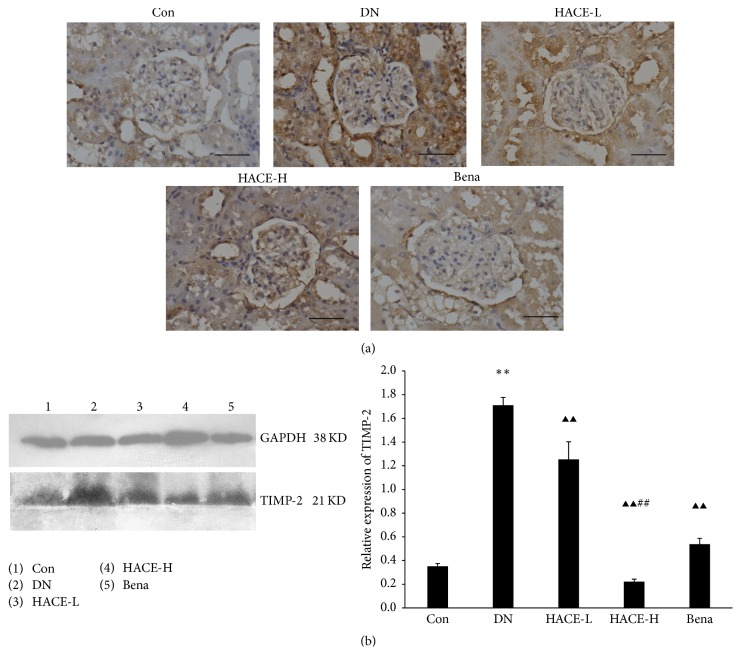
HACE suppressed the expression of TIMP-2 in kidney. (a) The expression of TIMP-2 by immunohistochemistry. Bar = 100 *μ*m. (b) The expression of TIMP-2 by Western blot. GAPDH was shown as a loading control. The relative expression was the ratio of TIMP-2 to GAPDH conducted by densitometric analysis. Con: nondiabetic control group; DN: streptozotocin-induced diabetic group; HACE-L: low-dose-HACE-treated group; HACE-H: high-dose-HACE-treated group; Bena: benazepril. ^*∗∗*^*P* < 0.01 versus Con; ^▲▲^*P* < 0.01 versus DN; ^##^*P* < 0.05 versus Bena.

Tissue inhibitor of metalloproteinases-2 (TIMP-2) is the main inhibitor of MMP-2; further it can inhibit ECM degradation [11, 13]. To evaluate the effect of HACE on TIMP-2, the expression of TIMP-2 in glomeruli was determined by immunohistochemistry. Consequently, the expression level of TIMP-2 was significantly upregulated in the glomeruli of DN rats compared with the control group; then the increased expression was strikingly reduced by high-dose HACE and positive drug benazepril (Figure 5(a)). Subsequently, it was further confirmed by the results of Western blot in the present study; that is, the protein level of TIMP-2 (Figure 5(b)) was evidently upregulated in the renal cortex of DN rats compared with control group (*P* < 0.01) but was distinctly inhibited by high-dose HACE and benazepril compared with the DN group (*P* < 0.01), and high-dose HACE treatment had better effect than benazepril treatment (*P* < 0.01). Therefore, HACE treatment could inhibit the expression of TIMP-2.

### 3.6. HACE Inhibited the Expression of PAI-1 in Glomeruli of Rats with DN

**Figure 6:**
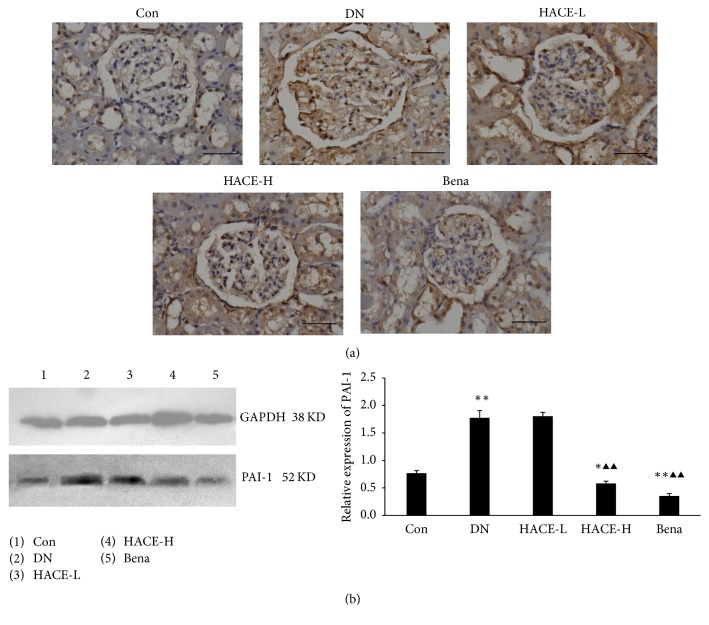
HACE suppressed the expression of PAI-1 in kidney of rats with diabetic nephropathy. (a) The expression of PAI-1 by immunohistochemistry. Bar = 100 *μ*m. (b) The expression of PAI-1 by Western blot. GAPDH was shown as a loading control. The relative expression was the ratio of PAI-1 to GAPDH conducted by densitometric analysis. Con: nondiabetic control group; DN: streptozotocin-induced diabetic group; HACE-L: low-dose-HACE-treated group; HACE-H: high-dose-HACE-treated group; Bena: benazepril. ^*∗*^*P* < 0.05 versus Con; ^*∗∗*^*P* < 0.01 versus Con; ^▲▲^*P* < 0.01 versus DN.

The activation of MMP required plasminogen activator, but plasminogen activator inhibitor-1 (PAI-1) inhibited the plasminogen activator [15, 17]. In the present study, the expression of plasminogen activator inhibitor-1 (PAI-1) in glomeruli was determined by immunohistochemistry. Accordingly, the expression of PAI-1 was significantly upregulated in the glomeruli of DN rats compared with the control group; then the increased expression was strikingly reduced by high-dose HACE-H and benazepril treatment (Figure 6(a)). This indicated that HACE could inhibit the expression of PAI-1 in glomeruli of DN rats. Following that, the results were further confirmed by Western blot analysis; that is, the protein level of PAI-1 (Figure 6(b)) was evidently upregulated in the renal cortex of DN rats compared with the control group (*P* < 0.01) but was distinctly inhibited by high-dose HACE-H and benazepril (Figure 6(b), *P* < 0.01). Therefore, HACE treatment inhibited the expression of PAI-1 in glomeruli of DN rats.

### 3.7. HACE Inhibited the Expression of tTG in Glomeruli of Rats with DN

**Figure 7:**
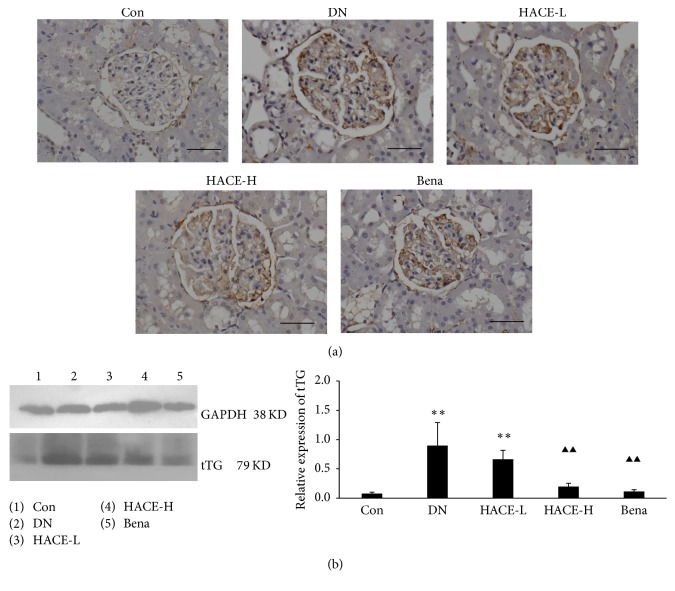
HACE treatment regulated the expression of tTG in glomeruli of rats with diabetic nephropathy. (a) The expression of tTG by immunohistochemistry. Bar = 100 *μ*m. (b) The expression of tTG by Western blot. GAPDH was shown as a loading control. The relative expression was the ratio of tTG to GAPDH conducted by densitometric analysis. Con: nondiabetic control group; DN: streptozotocin-induced diabetic group; HACE-L: low-dose-HACE-treated group; HACE-H: high-dose-HACE-treated group; Bena: benazepril. ^*∗∗*^*P* < 0.01 versus Con; ^▲▲^*P* < 0.01 versus DN.

Tissue transglutaminase (tTG) could lead to the accumulation of ECM [20]; this study found that the expression of tTG in renal cortex of rats was significantly upregulated in the glomeruli of DN rats compared with the control group by immunohistochemistry (Figure 7(a)); then the increased expression was strikingly reduced by high-dose HACE and benazepril treatment (Figure 7(a)). Then, Western blot analysis further confirmed that the protein level of tTG (Figure 7(b)) was evidently upregulated in the renal cortex of DN rats compared with the control group (*P* < 0.01) but was distinctly inhibited by high-dose HACE and benazepril (Figure 7(b), *P* < 0.01). Therefore, HACE inhibited the expression of tTG in glomeruli of rats with DN.

## 4. Discussion

Diabetic nephropathy (DN) like any other chronic diabetic complications is caused by various reasons, including poor glycemic control [28], high blood pressure, and high cholesterol (especially hypertriglyceridemia) [26]. In this study, it was found that herba artemisiae capillaris extract (HACE) treatment could decrease the level of blood glucose and lipid, and the results were consistent with Pan et al.'s study [25]; therefore HACE could control the DN inducements to inhibit the development of DN disease. Meanwhile it was reported that renal extracellular matrix (ECM) agglomeration was one of pathological characters of DN [29], and this study found that HACE treatment could inhibit the ECM accumulation and decreased the expression of fibronectin and type IV collagen, which were the components of ECM; therefore HACE treatment could inhibit renal pathological alteration at the DN stage. Herba artemisiae capillaris is used to alleviate liver fibrosis characterized by the excessive accumulation of ECM [21, 23, 30]; considering this study, it was concluded that HACE treatment could inhibit ECM accumulation in kidney and liver to protect those tissues from damage. Furthermore, HACE treatment decreased the level of albuminuria of DN rats, indicating that HACE treatment could improve the renal function of DN rats. Therefore, it was concluded that HACE could decrease the level of blood glucose and lipid to improve renal structure and function of DN rats.

By the way, in this study, we used the classical streptozotocin-induced DN model [31–34]. In this model, streptozotocin increased the level of blood glucose and the level of lipid (including low-density lipoprotein-cholesterol and total cholesterol), which was consistent with previous study [35, 36]. Meanwhile, in this model, streptozotocin did not affect the level of blood urea nitrogen obviously compared to control group, and HACE treatment groups were the same. Moreover, in this model, high-dose HACE treatment obviously decreased the level of triglyceride compared to control group, indicating that HACE treatment might have more potential than benazepril. Benazepril has been considered as a classic treatment drug for DN, which showed outstanding improvement of DN by decreasing the level of urinary albumin, promoting creatinine clearance, and reducing the pathological damage of the renal tissues closely related to reducing the expression of TGF-*β*1 and Nephrin [37–39], and this study confirmed that benazepril could decrease the level of albuminuria and total cholesterol and decrease the ECM accumulation in DN rats, which was consistent with previous study [25]. At the same time, it was noted that low-dose HACE has no striking reducing efficiency of blood glucose, while high-dose HACE showed more reduction of blood glucose than DN group. Therefore, we should pay attention to the appropriate dose of HACE in diabetic patients and should carry out further experiments before medical application of HACE.

Then, the mechanism of HACE preventing the development of streptozotocin-induced DN in rat from the point of decreasing the EMC accumulation was analyzed; the main systems degrading content of ECM majorly consist of matrix metalloproteinases (MMPs) and tissue transglutaminase (tTG) [11]. Under DN state, plasminogen activator inhibitor-1 (PAI-1) is upregulated to inhibit the plasminogen activities and thus decrease the activities of plasmin and further subsequently decrease the activates of plasmin-dependent MMPs [15, 16, 18]. Moreover, tissue inhibitor of metalloproteinases (TIMPs) can inhibit the activities of MMPs by noncovalent bonding to the activated MMPs on the proportion of 1 : 1, and TIMPs can also inhibit the activation of MMP by combining with plasminogen [17]. In this study, MMP-2 expression was decreased, PAI-1 and TIMP-2 expressions were increased in DN group compared to control group, and they were reversed by HACE or benazepril treatment, respectively; therefore it was concluded that upregulated PAI-1 and TIMP-2 inhibited the activity and expression of MMP-2 and then led to the ECM accumulation in streptozotocin-induced DN model, and HACE treatment inhibited ECM accumulation through regulating MMP-2, PAI-1, and TIMP-2 expressions. Apart from MMPs, tTG is also a key factor to ECM accumulation in DN; it can couple with fibronectin, collagen, and collagen peptide through covalent cross-linking effect, leading to the widespread of ECM proteins which promote ECM accumulation [40]; in this study, it was found that tTG expression was upregulated in DN group compared to control group, and HACE treatment could decrease the expression of tTG. Therefore, HACE inhibited ECM accumulation of DN rats through regulating the expression of MMP-2 and tTG.

Kidney is abnormally sensitive to oxidative stress; under the condition of high glucose, reactive oxygen species (ROS) can induce renal cell apoptosis and drop from basement membrane, causing damaged glomerular filtration membrane integrity and even proteinuria; moreover, ROS will activate the signal transduction system of ECM protein synthesis in the cells, thereby promoting the development of DN [41]. There are mainly several kinds of antioxidant defense system, including glutathione peroxidase (GSH-PX), hydrogen peroxidase (CAT), and superoxide dismutase (SOD) [42]. In this study, the activities of GSH-PX, SOD, and CAT and the content of MDA in serum and glomeruli tissue for analyzing systemic and local ability to clean ROS were detected. Compared with control group, the activity of GSH-PX in glomeruli and CAT in glomeruli was decreased in streptozotocin-induced diabetic nephropathy of rats; therefore the ability of scavenging ROS in local kidney was destroyed in DN model, which was further confirmed by increased level of glomerulus malondialdehyde (MDA) which is another important indicating index of oxidative stress* in vivo* [28], but, after HACE treatment, the activity of GSH-PX in glomeruli was obviously increased, and the level of MDA was obviously decreased; meanwhile it was noted that SOD activity in glomeruli was significantly increased, and CAT activity had an increasing trend; therefore HACE treatment was promoted to scavenge ROS in kidney through regulating enzymatic activity. Meanwhile HACE increased the activity of GSH-PX in serum and CAT in serum, but benazepril could not, indicating that HACE could clean systemic reactive oxygen species better than benazepril. Actually the major constituents of HACE were phenolic acids, coumarins, flavonoids, and 4-hydroxyacetophenone, which have been known to have pharmacological activity against oxidative stress [43, 44]; therefore those studies were consistent with our study.

## 5. Conclusion

In the present study, DN development was demonstrated in the streptozotocin-induced rats, and HACE treatment inhibited the DN progress through inhibiting the ECM accumulation and cleaning ROS. Therefore, HACE might be a promising drug for DN therapy.

## Figures and Tables

**Table 1:** The value of blood glucose, blood lipid, and indicators of renal function after HACE treatment.

	Con + Con	DN + Con	DN + HACE-L	DN + HACE-H	DN + Bena
Glucose (mmol/L)	8.20 ± 1.35	24.07 ± 11.93^*∗∗*^	30.43 ± 0.93^*∗∗*#^	15.08 ± 11.80^*∗*▲^	19.67 ± 8.43^*∗*^
BUN (*μ*mol/L)	4.19 ± 0.46	9.58 ± 4.18	10.10 ± 4.62	10.03 ± 5.62	11.43 ± 6.76^*∗*^
CREA (*μ*mol/L)	36.60 ± 2.73	31.30 ± 10.60	39.80 ± 0.52	34.10 ± 5.79	38.15 ± 4.52
Albuminuria (mg)	0.20 ± 0.11	0.50 ± 0.15^*∗*^	0.45 ± 0.21^*∗*^	0.34 ± 0.15^*∗*▲^	0.34 ± 0.13^*∗*▲^
T-CHO (mmol/L)	1.84 ± 0.18	2.20 ± 0.48^*∗*^	1.93 ± 0.54	1.61 ± 0.22^▲▲^	1.66 ± 0.15^▲▲^
TG (mmol/L)	1.09 ± 0.34	0.99 ± 0.59	0.72 ± 0.31	0.44 ± 0.19^*∗∗*▲^	0.87 ± 0.74
LDL-C (mmol/L)	0.36 ± 0.05	0.62 ± 0.22^*∗∗*^	0.51 ± 0.08	0.49 ± 0.12	0.48 ± 0.12
HDL-C (mmol/L)	1.18 ± 0.18	1.15 ± 0.31	1.21 ± 0.49	0.92 ± 0.25	0.94 ± 0.17

Twelve weeks after HACE treatment, blood glucose, blood lipid, and renal function parameters of the nondiabetic control group (Con), streptozotocin-induced diabetic group (DN), and the low-dose-HACE-treated (HACE-L) and high-dose-HACE-treated (HACE-H) and benazepril (Bena) positive control groups were tested. Except albuminuria in urine and blood glucose in plasma, other indicators were detected using the blood serum. BUN: blood urea nitrogen; CREA: creatinine; T-CHO: total cholesterol; TG: triglyceride; LDL-C: low-density lipoprotein-cholesterol; HDL-C: high-density lipoprotein-cholesterol. Data are presented as mean ± SD from 10 animals (*n* = 10) for each group. ^*∗*^*P* < 0.05 versus Con; ^*∗∗*^*P* < 0.01 versus Con; ^▲^*P* < 0.05 versus DN; ^▲▲^*P* < 0.01 versus DN; ^#^*P* < 0.05 versus Bena.
